# Coal

**Published:** 1857-09

**Authors:** 


### COAL.

We very much wish that poor people would patronize our Journal. We had rather have ten thousand poor subscribers than as many rich ones, because our object is to abate disease, and poverty is the principal cause of vice and crime, and sickness and death. Thus it is that we have so often directed the attention of our readers to the economics of life, and to the practice of those things which prevent thriftlessness and waste, and their consequent destitution. Besides, the poor need all the aid that can be given them in their hard struggle for bread; and we consider that we do a greater favor to humanity by teaching them how to avoid want, and thus helping them to stand upright, than in supplying those wants in the most princely manner, after they have fallen, for *having fallen, few ever rise again!* as every observant keeper of our city prisons will any day tell you. The poor are victimized at almost every step they take in our large cities: the penny swindle is a crying example, practised hourly in every block in New York. Cheated are they in the quantity and quality of every pound of meat they purchase, of every peck of vegetables. Thus it is that they who hardest earn their money, get the least value for it on its expenditure. Quite as cruel as any of these are the impositions practised on the poor in their purchases of coal. In the first place, there is that of short weight, which very few have the means of detecting. New York coal dealers, in purchasing coal, get twenty-two hundred and forty pounds for a ton; in retailing it, they never pretend to give more than two thousand pounds: how many of them fail to give even that, we do not pretend to say. A very effectual stop may be put to this shameful fraud, by the adoption of *Martin's self-weighing cart*, as sold by Mr. Olwine, of 377 Water street, New York. It would be a humanity, for every citizen to refuse taking coal from any other kind of cart, or to adopt some other equally efficient mode of securing full weight.

But there is a class of retail coal dealers, like advertising doctors, and patent medicine men, who would never become known for their virtues through ordinary channels, but call the newspapers to their aid, and, with a great show of honesty, guarantee full weight, or forfeit the coal. If coal were coal always, that would be fair enough, but advantage is taken of the unwary purchaser as follows: Of two carts of coal, standing side by side, an ordinary housekeeper would have no choice, while a practised coal dealer would gladly take a cargo of one while he would not be hired to admit the other into his yard, if compelled to send it to his best customers. How a poor man may tell one from the other with absolute certainty and instantly, is the object of this article, because one kind will burn almost entirely up, leaving but little residue except a few ashes, while in a single day's burning, the other will leave the grate nearly, if not quite full of rocks. One kind has only fifteen per cent. waste, the other has fifty or sixty. So one ton of coal, at one dollar a ton, may be dearer than another at four dollars, and yet, not one man in fifty could tell the difference. We have never seen that difference described in print. And much of the information contained in our pages is of this sort, as far as intended for popular enlightenment.

A shiny square fracture is what an honest coal dealer loves to see. He considers the article good in proportion as it breaks at right angles firmly. If it shatters in breaking, or breaks unsquarely, he will not look at it. If the coal has among it flat pieces, with a dull, coal-dust look, it is "bony." Such a piece gives no more heat than a bone: it is a black rock, nothing more; it is hard to kindle, and goes out directly. So, to give in a few words, the best idea of a good or bad load of coal, we say, the more square the lumps of coal are, the better it is. A

good quality of Red Ash coal, such as is almost universally burned in New York grates, has not a dozen pieces of flat coal in a whole load; the pieces are "*as broad as they are long.*" On the other hand, the more flat pieces there are, the more worthless is the article as fuel.

We have been speaking of what is called Anthracite coal, the kind that the generality of the people in New York use to burn in the grate.

The coal most highly prized to burn in New York, for common use, is called "*Red Ash*" coal, because the ashes it leaves in the grate are of a reddish color; and observation has shown that such a coal gives out the most heat, and leaves the least waste. Our range coal is called "White Ash," because the ashes it leaves are very much the color of wood ashes. It is used in stoves and in ranges for cooking purposes, because, being subject to a greater draft than in a common grate, it "clinkers" less than the Red Ash would, and, at the same time, gives out a more intense heat. By clinkering less is meant, that the *White Ash* coal leaves behind it less of that hard, molten-looking refuse, than the Red Ash would, if exposed to the same draft.

But there is a kind of coal *called* "Red Ash," and sold as such, between which and the real *Red Ash*, there is as much difference as there is between the same article of goods in Broadway and the Bowery. It is really a pink ash, not gray, like wood ashes, nor decidedly red as the real Red Ash. The Pink Ash is scarcely distinguishable from a wood ash at a casual glance. The Red Ash is more of the color of the cover of the June number of our journal, or of the cover of the first six numbers in one, of this year. It is not much redder than the palm of the hand—than common sand-paper. Some might say it approximates to the color of iron rust, having only here and there a flake of white ash, when spread out on the hand as we do flour, when we look at it.

As there is full fifty per cent. difference in the coal we burn, and as it costs an ordinary family nearly a hundred dollars a year to supply it with coal, we have thought the space we have given to the subject will well repay our readers, especially as this is about the time for laying in the winter's supply of fuel.

If any of our readers were to ask us how to insure them the cheapest and best coal for their money, we would say to them, go to such men as Randolph and Skidmore, Henry Reeves, the brothers Truslow, Thurston, and men of that character, who got a good custom and wealth by selling a good article of coal, and kept that custom by continuing to do the same thing, and tell them to send you *from their yard* a certain quantity of *Red Ash coal.* You will then pay the highest price, but then you will get full weight, the cleanest article, and the best of its kind. You may in rare cases, get it for seventy cents less a ton, by taking it from the boats of another class of dealers, but by neglect of screening, by its bony nature, and its grayish instead of reddish ash, you lose in the end, at least double what you supposed you were saving. We mention a few names, not because there are not others just as good, not because of any partiality to the men named, or even because we know them by sight, but simply because we wanted to be definite, and had private means of knowing the qualities of coal in which these men have been in the habit of dealing.

While speaking of coal, which is a particularly black subject to us, it may be farther interesting to state, especially for the comfort of our other half, (and those like her,) who are afraid all the coal will be burned, and the world will one of these days freeze up, that although the steam frigate *Niagara*, when moving ten miles an hour, consumes in that hour very nearly two tons of coal, that is, in exact numbers, four thousand four hundred and eighty pounds, there is in England alone enough coal to last seventeen hundred years, at the rate she is now consuming it.

Not many families in New York consume more than twenty tons of coal in a year, but a single ocean steamer consumes twice that much in twenty-four hours. To carry a Collins steamer from New York to Liverpool, requires eight hundred tons of coal—enough to keep an ordinary family forty years, and a thousand steamers leave the port of New York alone every year.

Coal beds are measured like anything else, by their length, breadth, and thickness. A bed of coal, measuring three feet every way, yields a ton of coal: thus it is found that while England has enough to last her seventeen hundred years—or eight thousand one hundred and thirty-nine square miles, the

United States alone has one hundred and thirty-three thousand square miles. Missouri alone can furnish a hundred million tons of coal a year, for the next thirteen hundred years, so we are safe from freezing for the next two thousand years, and by that time Mr. Payne, or some other genius, will have set the river on fire, and commenced burning up the Atlantic.

Coal is supposed to be the product of vegetation kept under an immense pressure for countless ages. Anthracite coal (such as the eastern part of Pennsylvania yields,) is Bituminous coal (such as comes from Pittsburgh, Cumberland, and Liverpool) without the gas—gas-light of cities being made from Bituminous coal, obtained from the places just named, the Anthracite being by a longer pressure, with perhaps other agencies not yet known, deprived of the gas. The supply of Red Ash coal is less than that of any other, confined, as far as we yet know, to a limited territory, about a hundred miles from Philadelphia; and yet the Philadelphians seldom use it—they prefer the harder kind of Anthracite, because it gives a greater heat. New Yorkers prefer the Red Ash coal from the Schuylkill, because, having some flame, it makes a more cheerful fire, because it kindles easier, and does not make such a dry heat.

